# Projected growth of the adult congenital heart disease population in the United States to 2050: an integrative systems modeling approach

**DOI:** 10.1186/s12963-015-0063-z

**Published:** 2015-10-15

**Authors:** Catherine P. Benziger, Karen Stout, Elisa Zaragoza-Macias, Amelia Bertozzi-Villa, Abraham D. Flaxman

**Affiliations:** Department of Medicine, University of Washington, Seattle, WA USA; Department of Cardiology, University of Washington, Seattle, WA USA; Institute for Health Metrics and Evaluation, University of Washington, Seattle, WA USA

**Keywords:** Heart defects, Congenital, Mortality, Vital statistics, Survival, Population

## Abstract

**Background:**

Mortality for children with congenital heart disease (CHD) has declined with improved surgical techniques and neonatal screening; however, as these patients live longer, accurate estimates of the prevalence of adults with CHD are lacking.

**Methods:**

To determine the prevalence and mortality trends of adults with CHD, we combined National Vital Statistics System data and National Health Interview Survey data using an integrative systems model to determine the prevalence of recalled CHD as a function of age, sex, and year (by recalled CHD, we mean positive response to the question “has a doctor told you that (name) has congenital heart disease?”, which is a conservative lower-bound estimate of CHD prevalence). We used Human Mortality Database estimates and US Census Department projections of the US population to calculate the CHD-prevalent population by age, sex, and year. The primary outcome was prevalence of recalled CHD in adults from 1970 to 2050; the secondary outcomes were birth prevalence and mortality rates by sex and women of childbearing age (15–49 years).

**Results:**

The birth prevalence of recalled CHD in 2010 for males was 3.29 per 1,000 (95 % uncertainty interval (UI) 2.8–3.6), and for females was 3.23 per 1,000 (95 % UI 2.3–3.6). From 1968 to 2010, mortality among zero to 51-week-olds declined from 170 to 53 per 100,000 person years. The estimated number of adults (age 20–64 years) with recalled CHD in 1968 was 118,000 (95 % UI 72,000–150,000). By 2010, there was an increase by a factor of 2.3 (95 % UI 2.2–2.6), to 273,000 (95 % UI 190,000–330,000). There will be an estimated 510,000 (95 % UI: 400,000–580,000) in 2050. The prevalence of adults with recalled CHD will begin to plateau around the year 2050. In 2010, there were 134,000 (95 % UI 69,000–160,000) reproductive-age females (age 15–49 years) with recalled CHD in the United States.

**Conclusion:**

Mortality rates have decreased in infants and the prevalence of adults with CHD has increased but will slow down around 2050. This population requires adult medical systems with providers experienced in the care of adult CHD patients, including those familiar with reproduction in women with CHD.

**Electronic supplementary material:**

The online version of this article (doi:10.1186/s12963-015-0063-z) contains supplementary material, which is available to authorized users.

## Background

Congenital heart disease (CHD) is one of the most common types of congenital malformations in the United States (US), estimated to be between four and nine per 1,000 births, and without surgery it is are often incompatible with long-term survival [1–6]. Patients with CHD can have mild disease with relatively little need for medical care; however, others have complicated physiology and require specialized care. One study found the birth prevalence of moderate and severe CHD to be about six per 1,000 births (and increases to 19 per 1,000 if serious bicuspid aortic valves are included) [4]. With improved surgical techniques and increased neonatal screening, the mortality in CHD has shifted from the neonatal period into adulthood with a growing population of adults with CHD [7–12]. However, the mortality in CHD is still premature compared to the general population and varies by socioeconomic status and race [9, 11]. A recent systematic review estimated adults with CHD to be around 3,000 per million [13] but this estimate is not specific to the US. In addition, predictions about the growth of this population over time are lacking. This is important information for the healthcare system because these patients are not cured and they will continue to have excess mortality and suffer complications related to their CHD and surgical procedures during their adult life [14, 15].

Because adults with CHD are a new patient population, medical providers and care systems have relatively little expertise with the unique issues of adult CHD patients. These patients are complex not only because of their CHD, but unique to this population is the interaction between CHD and issues of young adulthood, including reproduction, neurodevelopmental abnormalities, and genetic syndromes for which CHD is only one part of the phenotypic expression [16]. Thus, the expertise found in pediatric medicine that has successfully treated these patients for decades now must be developed in adult medicine. Guidelines recommend that all adults with moderate to severely complex CHD be evaluated by adult CHD experts [17], which are currently few in number and insufficient for the growing adult CHD population [18]. However, many adult CHD patients, families, and medical providers are unaware of the need for continued lifelong subspecialty follow-up, especially in those with moderate to severe disease [19–21].

Thus, to ensure the highest quality care for these complex and unique patients, more accurate estimates of the adult population with moderate to severe CHD are needed, as well as future projections to help inform the health care system [22].

We used integrative systems modeling [23],which combines a mechanistic model of disease progression with a statistical model of data likelihood, to simultaneously estimate the prevalence and mortality of CHD in the US from multiple data sources. From this model, we made age-/sex-specific estimates of CHD population size from 1968 to 2010, and projected future trends to 2050.

## Methods

### Data sources

#### CHD prevalence

The National Health Interview Survey (NHIS) has annually asked about CHD status in a large sample of the US population [24]. Starting in 1997, it has included the following question for a random sample of individuals younger than 18: “Looking at this list, has a doctor or health professional ever told you that (selected child name) had any of these conditions? … (9) congenital heart disease?” We used positive response to this question for years 1997 to 2011 to calculate the sex-/year-specific measurements of prevalence for single-year age groups, which we referred to as “recalled CHD” and interpreted as a surrogate for the prevalence of moderate to severe CHD. Recalled CHD is a vast underestimate of the overall CHD prevalence but reflects the public knowledge of CHD and potential cases that will encounter the health care system. In order to answer yes to the question, the participant had to be familiar with the term “CHD” and remember that their child had a heart condition that is classified as a CHD. Recalled CHD is likely biased to those with more moderate and severe disease since these require multiple follow-up appointments or surgery. However, it is also possible that cases of simple CHD were captured by this question. To avoid age-differential nonresponse bias, we excluded age group zero from the primary analysis.

#### CHD mortality

The National Center for Health Statistics in the US has annually compiled data from all filed death certificates and has made these data available on its Multiple-Cause Mortality Files [25]. These files include demographic and geographic information on the decedent and International Classification of Disease (ICD) codes for the underlying cause of death and contributing factors on the death certificate. Three versions of the ICD were used to code causes of death in the mortality files (Additional file 1: Table S1). The selected range of codes includes congenital anomalies of the heart and great vessels and excludes anomalies of the peripheral circulatory system. We defined deaths associated with CHD if the records had any mention of such codes as a cause of death or if the code was listed in as an underlying cause of death. From this we tabulated sex-/year-specific counts of CHD deaths for single-year age groups for years 1968 to 2010.

#### Population size

We used the Human Mortality Database (http://www.mortality.org/) and US Census Department 2012 Middle Series projections to determine age-/sex-/year-specific US population. We obtained CHD mortality rate data for each age/sex/year group by dividing the CHD mortality counts by the midyear population for each group.

The age-/sex-/year-specific data on CHD mortality rate and CHD prevalence were used as inputs into our statistical model of disease rates as function of age and time [26]. We used parametric bootstrap resampling of the input data to generate 95 % uncertainty intervals (UI). We multiplied the appropriate population estimates by the estimated prevalence to produce estimates of the size of the CHD-prevalent population by age, sex, and year.

Age- and sex-specific prevalence and with-condition mortality rates for each year were calculated simultaneously for all time periods (separately for males and females). We examined trends in age-specific with-condition mortality rates between 1968 and 2010 by year of death, pooled across sexes. We present the data using the following age groups: 0–51 weeks, 1–4 years, 5–9 years, 10–14 years, 15–19 years, and 20–65 years.

### Modeling

**Fig. 1:**
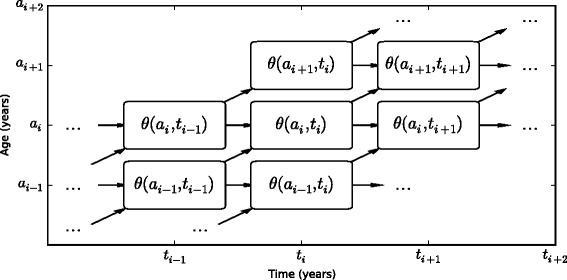
Integrative systems model combines mortality and prevalence data in a non-linear regression framework, based on an age- and time-specific compartmental model of disease progression

We used the integrative systems model DisMod-PDE to combine the prevalence and mortality data using a compartmental model of process and an offset log-normal model of data [26]. DisMod-PDE is a nonlinear regression model that uses a system of differential equations to relate age- and time-specific progression of disease through a two-compartment model. When modeling a congenital condition such as CHD, the model simplifies to include only the age-/time-specific excess-mortality hazard (*χ*) quantifying the flow out of the with-condition compartment (*C*) and the time-specific birth prevalence of the condition (*C*(0,*t*)). For more detail, see Additional file 2. DisMod-PDE is a Bayesian model, and we used weakly informative priors to allow the data to inform the estimates as much as possible (Fig. 1). Stock in compartment *C* was assumed to vary over age and time and was smoothed across cohorts with second-order smoothing of *σ* = 1. Hazard *χ* was assumed to also vary over age and time and to have second-order smoothing across ages, cohorts, and cross-smoothing with *σ* = 1. We used a deterministic differential equation restricted to only compartment *C* and hazard *χ* of DisMod-PDE. Additional assumptions of the model are the following: 1. there was no incidence besides at birth; 2. there was no remission (no one was cured from the condition); and 3. there was no protective effect associated with the condition. We also assumed that the birth prevalence rate was constant over time, and that the prevalence rate and excess mortality rate were constant across cohorts before 1968. We conducted a sensitivity analysis to quantify the influence of our assumptions; see Additional file 3. When making projections, we conservatively assumed that the birth prevalence and excess mortality rate were constant across cohorts after 2010, as well. We were unable to distinguish between disease severity based on the NHIS data and therefore we estimate a single (age- and time-specific) prevalence rate and excess mortality rate for all recalled CHD. Analysis was undertaken using DisMod-PDE software, with additional processing done with Python 2.7 [26, 27]. A replication archive is available online (http://ghdx.healthmetricsandevaluation.org/record/united-states-adult-congenital-heart-disease-estimates-1970-2050).

## Results and discussion

### CHD mortality

**Fig. 2:**
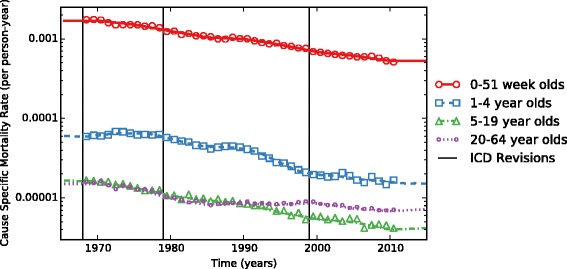
Congenital-heart-disease-specific mortality rates as a function of time from 1970 to 2010, stratified by age group. (Markers show National Vital Statistics System data and lines show model estimates.)

The multiple-cause mortality files for all ages for 1968 to 2010 contain records for 92.4 million deaths by any cause. We identified 288,813 deaths (0.31 %) associated with a CHD. In 73.4 % of these deaths (212,116, or 0.23 % of all), the CHD was the underlying cause, i.e., the death was coded as due to a CHD. Between 1968 and 2010, the cause-specific mortality rate for all ages declined 71 %, from 4.9 to 1.4 per 100,000 person-years (PY) (Fig. 2). The mortality was higher among males (1.5 per 100,000) than among females (1.3 per 100,000). Among zero to 51-week-olds, it declined 69 % whereas among adults aged 20–64 years, the mortality declined 55 %. The percentage of CHD deaths that occur during the first year of life has declined from 61 % in 1968 to 46 % in 2010.

### CHD prevalence

**Fig. 3:**
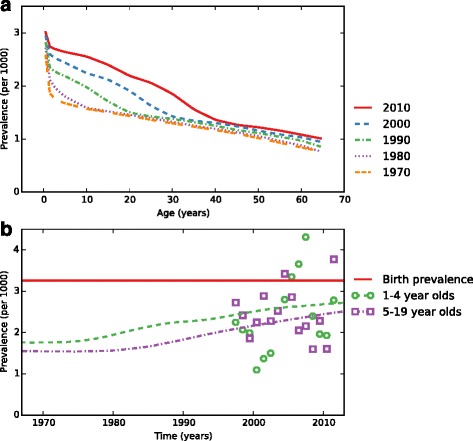
**a** Recalled congenital heart disease prevalence per 1000 as a function of age, stratified by year of birth. **b** Recalled prevalence per 1000 at birth (solid red), for 1–4 year olds (dashed green), and for 5–19 year olds (dotted purple) as a function of time, based on data from the National Health Interview Survey (circle and square markers)

The NHIS data from 1997 to 2011 contains recalled CHD status for 180,766 individuals, from which our model estimates that the birth prevalence of recalled CHD in 2010 for males was 3.29 per 1,000 (95 % UI 2.8–3.6), and for females was 3.23 per 1,000 (95 % UI 2.3–3.6) (Fig. 3b).

### Prevalence of adult congenital heart disease

Figure 3a shows the prevalence of recalled CHD over time by age group. In 1968, there were 118,000 (95 % UI 72,000–150,000) adults with recalled CHD. By 2010, there was an increase by a factor of 2.3 (95 % UI 2.2–2.6) to 273,000 (95 % UI 190,000–330,000) adults. In 2010, there were 134,000 (95 % UI 69,000–160,000) reproductive-age females (age 15–49 years) with recalled CHD.

**Fig. 4:**
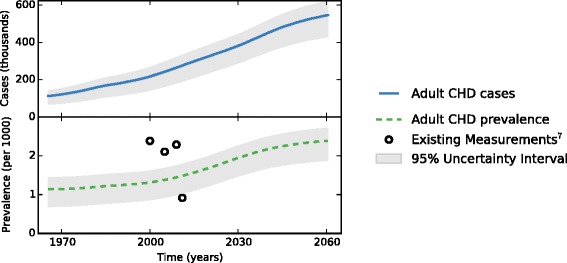
Estimated number of adults (ages 20 to 64 years) with recalled congenital heart disease cases (blue solid line) and prevalence of recalled congenital heart disease in adults (per 1000) (green dotted line), with 95 % uncertainty intervals shaded in grey, as a function of time, from 1965 to 2060. Previous estimates of ACHD prevalence are marked with circles for comparison

The estimated number of adults (age 20 to 64 years) with recalled CHD, as well as the trends in prevalence from 1970 to 2050, are shown in Table 1 and displayed visually in Fig. 4. There will be an estimated 355,000 (95 % UI: 266,000–415,000) adults age 20 to65 years with CHD in 2025 and 510,000 (95 % UI: 400,000–580,000) in 2050. This corresponds to an estimated 1.47 per thousand adults with CHD in 2010, 1.83 per thousand adults in 2025, and 2.31 per thousand adults in 2050. The prevalence of adults with recalled CHD begins to plateau around the year 2050 and growth in the total number of adults with CHD slows to match the general population growth.

**Table 1:** Estimated cases and prevalence of ACHD over time

	1970	1980	1990	2000	2010	2020	2030	2040	2050
ACHD Cases (Thousands)	122	154	183	219	273	328	385	454	510
ACHD Prevalence (per 1000)	1.15	1.19	1.25	1.32	1.47	1.7	1.96	2.18	2.31

In 2010, the number of adolescents with recalled CHD who turned 20 years old was 9,800 (95 % UI: 7,700–11,000). This number will increase by a factor of 1.29 to 13,000 (95 % UI: 10,000–14,000) in 2050. In addition, there will be 170,000 (95 % UI: 100,000–200,000) reproductive-age females in 2025 and 216,000 (95 % UI: 140,000–250,000) in 2050.

### Main findings

We found 273,000 adults had recalled CHD in 2010, which corresponds to 1.47 per thousand people. This estimate is lower than a recent meta-analysis, which found the adult CHD population prevalence to be around three per thousand, but they were limited by the large heterogeneity of the studies and did not include the US [13]. Our integrative systems model confirms that the population of adults with CHD is increasing; however, we are the first to note that the prevalence will start to plateau around the year 2050 unless there are significant changes in birth prevalence or mortality. This has implications for the health care system since it allows us to quantify the magnitude of this patient population and help with planning for their future healthcare needs. Previous observational studies of adults with CHD have focused on the severity distribution, and found that severe cases constitute 3 % and moderate cases 15 % of the total; none have projected future population trends [4, 13, 28]. Registry data are thus far lacking, and it is difficult to establish registries that capture adult CHD patients with heterogeneous diseases who may not receive regular healthcare [29]. Mathematical modeling has been recommended as an important tool for understanding the burden of this disease [22].

This study used integrative systems modeling to show that the mortality from CHD has declined in all age groups between 1968 and 2010 with children zero to 51 weeks having experienced the greatest decline, followed by those 1–4 years, which is consistent with prior studies that have shown a mortality decreased between 31 and 39 % in this population [2, 3, 30]. Despite improvements in mortality, these age groups continue to experience the highest mortality from CHD. Recent implementation of newborn pulse oximetry screening programs for critical CHD [31], as well as increased perinatal screening using genetic testing and fetal echocardiography, allows earlier detection of CHD. Earlier diagnosis will continue to decrease the infant mortality since late diagnosis is associated with worse infant survival [32, 33]. However, we still have much work to do in individuals aged 20 to 64 years old, whose mortality has remained stable with little improvement over the past 30 years [7]. Our data suggest that the mortality in CHD beyond the neonatal period is shifted well into adulthood, though still premature compared to the general population.

The care of adult CHD patients requires specialized training, which is being developed after the 2012 approval of adult CHD as a cardiology subspecialty by the American Board of Medical Specialties. The current American Heart Association and American College of Cardiology guidelines recommend that adults with moderate and severe CHD be seen every 12 to 24 months by a cardiologist with specific CHD expertise at a regional CHD center; the absence of symptoms is not a reliable indicator of cardiac function [17, 34–36]. The leading cause of death in the adult CHD population is sudden death (26 %), followed by progressive heart failure (21 %) and perioperative death (18 %) [34]. Unfortunately, less than half of adolescent patients have adult cardiology follow-up in a timely manner after they turn 18 years old [19, 37].

This rapidly increasing CHD population in adults, as well as the increasing population of atherosclerotic heart disease in adults [38], will lead to a substantial increase in health care utilization [39–41] and increase demand for CHD trained cardiologists. It also requires an estimated one specialized cardiac center per two million population, which are not yet in place [42]. In addition, CHD patients benefit from multi-disciplinary care team to address their complex needs given their increased risk for developmental disabilities [16], comorbidities [43], and special considerations for patients desiring pregnancy [44, 45]. Collaborative and multidisciplinary strategies are urgently needed, such as systems and processes to improve transitions of care from pediatric providers to the adult health care system [20, 37, 46], improved specialized cardiac centers with cardiologists trained in adult CHD management [42], and technological and medical improvements in care for these patients [42, 47].

### Limitations

Our data on CHD prevalence come entirely from the NHIS, a population-based household survey subject to all the challenges of survey research. Although NHIS has a high response rate (close to 90 %), it is possible that there is differential non-response bias, where households with children with moderate to severe CHD are more likely than average to refuse to be interviewed. We attempted to minimize the bias that this would introduce by excluding the data from those with children in the zero to 51 week age group, and sensitivity analysis (Additional file 3) showed that including it reduced the overall prevalence by at most 4 %. CHD is a group of individually rare diseases and so it is likely unrecalled or unreported when present (it is also sometimes undiagnosed or diagnosed later in life, making recall impossible). It is also possible that CHD is reported in cases where it is not present, due to confusion about the question. However, we believe that recalled CHD is an underestimate of the overall prevalence of CHD, which reflects the lack of public understanding, even among those with the condition. As the question only asked for children under the age of 18, it cannot capture CHD cases diagnosed in adulthood. The recency effect predicts that this subpopulation would better recall their CHD status than those diagnosed in childhood, but we suspect that they constitute a small proportion of all ACHD cases. Despite the large sample size of the NHIS, recalled CHD is rare enough that the data are quite noisy (Fig. 3b). Our results show that since 1997, there has been a birth prevalence of recalled CHD of around three per 1,000, which we would like to use as an estimate of moderate to severe cases of CHD. This is lower than recently published birth prevalence of four to seven per 1,000 [1, 3–5]. At birth, about half of all CHD are characterized as moderate to severe; in adulthood moderate CHD accounts for around 38 % and severe around 15 % [13, 17]. Therefore, as the aim of our study was to capture those aware of their moderate to severe CHD who would survive to adulthood, to determine the current and future population of adults that will enter the health care system, we feel this reflects the minimum number of adult CHD patients we would expect to engage in the health system unless increased public health awareness on the individual and population level are improved.

Our data on CHD mortality come from an ICD-coded nationwide database, which may have a low specificity due to miscoding. It may also be nonspecific [48] leading to under-reporting [49] or over-reporting [50]; however, the sensitivity of the system has remained largely the same so the time trends should still be valid. The mortality rates may be underestimates as the patient or family and provider need to be aware of the CHD diagnosis in order for it to be coded on the death certificate. We noted minor variations in the data between ICD 8 and 9 and between 9 and 10 but this did not change the mortality trends. An alternative line of research has used National Birth Defects Prevention Network (NBDPN) data to investigate the survival rates for (and birth prevalence of) specific defects [6, 9–12, 51]. This provides complementary information to our study, but, since it has been more focused in its cause lists (e.g. considering birth prevalence or survival for hypoplastic left heart syndrome only [52]), it cannot be incorporated directly into our model. Developing estimates and projections for more focused cause lists that bring together NBDPN measurements and death certificate data is an interesting direction for future work. We anticipate that future researcher will use other approaches and/or refine those used here to further add to our knowledge of the prevalence of ACHD.

Our finding of decreased infant mortality is consistent with other studies and is not likely due to a decrease in births, which was adjusted for in our model. Other possible explanations are also unlikely, but include a decreasing birth prevalence with an increasing diagnosis of CHD. However, published studies suggest the opposite, with slightly increased birth prevalence given the increase in the diagnosis with perinatal screening with fetal echocardiography and neonatal screening with pulse oximetry [1, 3, 5, 30, 53]. Also, birth prevalence may increase with increased number of adults with CHD having children with CHD [54]; however, this may be offset by increased termination of pregnancy due to high complex CHD malformations [55]. Data related to termination of pregnancy are limited and trends are unknown [56]. Thus, our findings of an increased population of adults with recalled CHD due to the decrease in mortality and increased life expectancy will lead to a growing population in the future.

## Conclusion

In this study we estimated and projected future prevalence and population trends of adults with recalled CHD using an integrative systems model. The use of death certificates, which record nearly all deaths, and NHIS population-based data, enabled us to assess the impact of CHD on essentially the entire population, not only those who undergo surgery or who are seen at specialized cardiac centers. We found a 3.2-fold decreased mortality in 0 to 51-week-olds and a rapidly increasing population of adults with CHDs that will start to plateau by the year 2050. There is a need for increased awareness and understanding of the term “CHD”, both individually and on the population level, with improved transitions from pediatric to adult care and an adult health care system that is prepared for the complex needs of this population.

## Additional files

Additional file 1: Table S1. ICD codes used. (DOC 107 kb) Additional file 2:
**DisMod-PDE model for ACHD.** (PDF 126 kb) Additional file 3:
**Sensitivity analysis of ACHD DisMod-PDE parameters.** (PDF 94 kb)

## Abbreviations

ACHD: Adult congenital heart disease
CHD: Congenital heart disease
US: United States
NBDPN: National Birth Defects Prevention Network
NHIS: National Health Interview Survey
ICD: International Classification of Diseases
