# Supplementary material for: Projected growth of the adult congenital heart disease population in the United States to 2050: an integrative systems modeling approach
Source: Popul Health Metr. 2015 Oct 15;13:29. doi: 10.1186/s12963-015-0063-z (PMC4606959; doi:10.1186/s12963-015-0063-z)
Supplement: Additional file 1: Table S1. — ICD codes used. (DOC 107 kb) [file 12963_2015_63_MOESM1_ESM.doc]

Online supplementary table 1: ICD codes used

| ICD Revision | Cause Number and Name |
| --- | --- |
| ICD 8 | 746 Congenital anomalies of heart |
| ICD 8 | 746.0 Common truncus |
| ICD 8 | 746.1 Transposition of great vessels |
| ICD 8 | 746.2 Tetralogy of Fallot |
| ICD 8 | 746.3 Ventricular septal defect |
| ICD 8 | 746.4 Atrial septal defect |
| ICD 8 | 746.5 Ostium atrioventriculare commune |
| ICD 8 | 746.6 Anomalies of heart valve |
| ICD 8 | 746.7 Fibroelastosis cordis |
| ICD 8 | 746.8 Other specified anomalies of heart |
| ICD 8 | 746.9 Unspecified anomalies of heart |
| ICD 8 | 747 Other congenital anomalies of circulatory system |
| ICD 8 | 747.0 Patent ductus arteriosus |
| ICD 8 | 747.1 Coarctation of aorta |
| ICD 8 | 747.2 Other anomalies of aorta |
| ICD 8 | 747.3 Stenosis or atresia of pulmonary artery |
| ICD 8 | 747.4 Anomalies of great veins |
| ICD 8 | 747.8 Other specified anomalies of circulatory system |
| ICD 8 | 747.9 Unspecified anomalies of circulatory system |
| ICD 9 | 745 Bulbus cordis anomalies and anomalies of cardiac septal closure |
| ICD 9 | 745.0 Common truncus |
| ICD 9 | 745.1 Transposition of great vessels |
| ICD 9 | 745.10 Complete transposition of great vessels |
| ICD 9 | 745.11 Double outlet right ventricle |
| ICD 9 | 745.12 Corrected transposition of great vessels |
| ICD 9 | 745.19 Other |
| ICD 9 | 745.2 Tetralogy of Fallot |
| ICD 9 | 745.3 Common ventricle |
| ICD 9 | 745.4 Ventricular septal defect |
| ICD 9 | 745.5 Ostium secundum type atrial septal defect |
| ICD 9 | 745.6 Endocardial cushion defects |
| ICD 9 | 745.60 Endocardial cushion defect, unspecified type |
| ICD 9 | 745.61 Ostium primum defect |
| ICD 9 | 745.69 Other |
| ICD 9 | 745.7 Cor biloculare |
| ICD 9 | 745.8 Other |
| ICD 9 | 745.9 Unspecified defect of septal closure |
| ICD 9 | 746 Other congenital anomalies of heart |
| ICD 9 | 746.0 Anomalies of pulmonary valve |
| ICD 9 | 746.00 Pulmonary valve anomaly, unspecified |
| ICD 9 | 746.01 Atresia, congenital |
| ICD 9 | 746.02 Stenosis, congenital |
| ICD 9 | 746.09 Other |
| ICD 9 | 746.1 Tricuspid atresia and stenosis, congenital |
| ICD 9 | 746.2 Ebstein's anomaly |
| ICD 9 | 746.3 Congenital stenosis of aortic valve |
| ICD 9 | 746.4 Congenital insufficiency of aortic valve |
| ICD 9 | 746.5 Congenital mitral stenosis |
| ICD 9 | 746.6 Congenital mitral insufficiency |
| ICD 9 | 746.7 Hypoplastic left heart syndrome |
| ICD 9 | 746.8 Other specified anomalies of heart |
| ICD 9 | 746.81 Subaortic stenosis |
| ICD 9 | 746.82 Cor triatriatum |
| ICD 9 | 746.83 Infundibular pulmonic stenosis |
| ICD 9 | 746.84 Obstructive anomalies of heart, NEC |
| ICD 9 | 746.85 Coronary artery anomaly |
| ICD 9 | 746.86 Congenital heart block |
| ICD 9 | 746.87 Malposition of heart and cardiac apex |
| ICD 9 | 746.89 Other |
| ICD 9 | 746.9 Unspecified anomaly of heart |
| ICD 9 | 747 Other congenital anomalies of circulatory system |
| ICD 9 | 747.0 Patent ductus arteriosus |
| ICD 9 | 747.1 Coarctation of aorta |
| ICD 9 | 747.10 Coarctation of aorta (preductal) (postductal) |
| ICD 9 | 747.11 Interruption of aortic arch |
| ICD 9 | 747.2 Other anomalies of aorta |
| ICD 9 | 747.20 Anomaly of aorta, unspecified |
| ICD 9 | 747.21 Anomalies of aortic arch |
| ICD 9 | 747.22 Atresia and stenosis of aorta |
| ICD 9 | 747.29 Other |
| ICD 9 | 747.3 Anomalies of pulmonary artery |
| ICD 9 | 747.4 Anomalies of great veins |
| ICD 9 | 747.40 Anomaly of great veins, unspecified |
| ICD 9 | 747.41 Total anomalous pulmonary venous connexion |
| ICD 9 | 747.42 Partial anomalous pulmonary venous connexion |
| ICD 9 | 747.49 Other anomalies of great veins |
| ICD 9 | 759.3 Situs inversus |
| ICD 10 | Q20 Congenital malformations of cardiac chambers and connexions |
| ICD 10 | Q20.0 Common arterial trunk |
| ICD 10 | Q20.1 Double outlet right ventricle |
| ICD 10 | Q20.2 Double outlet left ventricle |
| ICD 10 | Q20.3 Discordant ventriculoarterial connexion |
| ICD 10 | Q20.4 Double inlet ventricle |
| ICD 10 | Q20.5 Discordant atrioventricular connexion |
| ICD 10 | Q20.6 Isomerism of atrial appendages |
| ICD 10 | Q20.8 Other congenital malformations of cardiac chambers and connexions |
| ICD 10 | Q20.9 Congenital malformation of cardiac chambers and connexions, unspecified |
| ICD 10 | Q21 Congenital malformations of cardiac septa |
| ICD 10 | Q21.0 Ventricular septal defect |
| ICD 10 | Q21.1 Atrial septal defect |
| ICD 10 | Q21.2 Atrioventricular septal defect |
| ICD 10 | Q21.3 Tetralogy of Fallot |
| ICD 10 | Q21.4 Aortopulmonary septal defect |
| ICD 10 | Q21.8 Other congenital malformations of cardiac septa |
| ICD 10 | Q21.9 Congenital malformation of cardiac septum, unspecified |
| ICD 10 | Q22 Congenital malformations of pulmonary and tricuspid valves |
| ICD 10 | Q22.0 Pulmonary valve atresia |
| ICD 10 | Q22.1 Congenital pulmonary valve stenosis |
| ICD 10 | Q22.2 Congenital pulmonary valve insufficiency |
| ICD 10 | Q22.3 Other congenital malformations of pulmonary valve |
| ICD 10 | Q22.4 Congenital tricuspid stenosis |
| ICD 10 | Q22.5 Ebstein's anomaly |
| ICD 10 | Q22.6 Hypoplastic right heart syndrome |
| ICD 10 | Q22.8 Other congenital malformations of tricuspid valve |
| ICD 10 | Q22.9 Congenital malformation of tricuspid valve, unspecified |
| ICD 10 | Q23 Congenital malformations of aortic and mitral valves |
| ICD 10 | Q23.0 Congenital stenosis of aortic valve |
| ICD 10 | Q23.1 Congenital insufficiency of aortic valve |
| ICD 10 | Q23.2 Congenital mitral stenosis |
| ICD 10 | Q23.3 Congenital mitral insufficiency |
| ICD 10 | Q23.4 Hypoplastic left heart syndrome |
| ICD 10 | Q23.8 Other congenital malformations of aortic and mitral valves |
| ICD 10 | Q23.9 Congenital malformation of aortic and mitral valves, unspecified |
| ICD 10 | Q24 Other congenital malformations of heart |
| ICD 10 | Q24.0 Dextrocardia |
| ICD 10 | Q24.1 Levocardia |
| ICD 10 | Q24.2 Cor triatriatum |
| ICD 10 | Q24.3 Pulmonary infundibular stenosis |
| ICD 10 | Q24.4 Congenital subaortic stenosis |
| ICD 10 | Q24.5 Malformation of coronary vessels |
| ICD 10 | Q24.6 Congenital heart block |
| ICD 10 | Q24.8 Other specified congenital malformations of heart |
| ICD 10 | Q24.9 Congenital malformation of the heart, unspecified |
| ICD 10 | Q25 Congenital malformations of great arteries |
| ICD 10 | Q25.0 Patent ductus arteriosus |
| ICD 10 | Q25.1 Coarctation of aorta |
| ICD 10 | Q25.2 Atresia of aorta |
| ICD 10 | Q25.3 Stenosis of aorta |
| ICD 10 | Q25.4 Other congenital malformations of aorta |
| ICD 10 | Q25.5 Atresia of pulmonary artery |
| ICD 10 | Q25.6 Stenosis of pulmonary artery |
| ICD 10 | Q25.7 Other congenital malformations of pulmonary artery |
| ICD 10 | Q25.8 Other congenital malformations of great arteries |
| ICD 10 | Q25.9 Congenital malformation of great arteries, unspecified |
| ICD 10 | Q26 Congenital malformations of great veins |
| ICD 10 | Q26.0 Congenital stenosis of vena cava |
| ICD 10 | Q26.1 Persistent left superior vena cava |
| ICD 10 | Q26.2 Total anomalous pulmonary venous connexion |
| ICD 10 | Q26.3 Partial anomalous pulmonary venous connexion |
| ICD 10 | Q26.4 Anomalous pulmonary venous connexion, unspecified |

The ICD, Eighth Revision (ICD-8), was implemented in 1968, ICD Ninth Revision (ICD-9), was implemented in 1978, and ICD Tenth Revision (ICD-10), was implemented in 1999. We identified records that contained any of the codes listed above as the underlying cause of death or listed as contributing cause of death.
