# Supplementary material for: Projected growth of the adult congenital heart disease population in the United States to 2050: an integrative systems modeling approach
Source: Popul Health Metr. 2015 Oct 15;13:29. doi: 10.1186/s12963-015-0063-z (PMC4606959; doi:10.1186/s12963-015-0063-z)
Supplement: Additional file 2: — DisMod-PDE model for ACHD. (PDF 126 kb) [file 12963_2015_63_MOESM2_ESM.pdf]

Online supplementary file 1: DisMod-PDE model for ACHD

Abraham D. Flaxman

This online supplementary file describes in detail the DisMod-PDE model for estimating prevalence of congenital heart disease (CHD) in the United States. The model produces brings together data from the NHIS and NVSS and weakly-informative priors in a Bayesian framework to produce estimates over time and for a wide range of ages. This document follows the notation developed in [1], but, for the convenience of the reader, it is intended to be a self-contained treatment. In the author's opinion, this is an excellent introduction to the DisMod approach to age-period-cohort modeling, because the complexity of descriptive epidemiological modeling is greatly reduced in the case of a congenital condition like CHD, which has no remission and no incidence (besides at birth, which is called birth prevalence in the preferred nomenclature of CHD epidemiologists).

1 The DisMod-PDE model

As described in [1], the DisMod-PDE model employs a two-compartment systems dynamics model of the progression of disease through a population, where the stocks and flows are all dependent on both time and age. Let a denote age and t denote time, and let $S(a,t)$ and $C(a,t)$ denote the fraction of a cohort susceptible and with-condition for a specific disease. Furthermore, let $\iota(a,t)$ be the incidence hazard, $\rho(a,t)$ be the remission hazard, $\chi(a,t)$ be the excess-mortality hazard, and $\omega(a,t)$ be the background-mortality hazard.

In this notation, the system of differential equations for the the two compartment model is

$$\begin{aligned}\frac{dS(a+\tau,t+\tau)}{d\tau} &= -(\iota+\omega)S+\rho C;\\ \frac{dC(a+\tau,t+\tau)}{d\tau} &= \iota S-(\rho+\omega+\chi)C.\end{aligned}$$

To make computation tractable, the age/time-specific stocks and flows are parameterized by knots for user-specified cohorts and ages in a computational grid, and values for a and t between grid points are calculated via bilinear interpolation. Although it is possible to make the differential equations stochastic in this formulation, for our purposes here, we will always enforce equality of the S

and C values with the approximation solution to the differential equations for the linearly interpolates ι, ρ, χ, and ω values.

Descriptive epidemiological data is often noisier than one would expect from sampling error alone, while age/time-specific disease parameters are expected to vary smoothly. We incorporate these observations into the model by smoothing across cohorts and ages.

Smoothing across cohorts is implemented as a penalty on second-order differences of points in the computational grid. For example, for with-condition stock C, for age grid point a, for cohorts c_k, c_{k+1}, c_{k+2}, the log-prior is equal to

$$-\frac{1}{\sigma}\Bigg[\frac{\log(C(a, c_{k+2}+a)) - \log(C(a, c_{k+1}+a))}{c_{k+2}-c_{k-1}} - \frac{\log(C(a, c_{k+1}+a)) - \log(C(a, c_k+a))}{c_{k+1}-c_k}\Bigg]^2,$$

where σ is the prior on second-order smoothing of C with respect to cohort.

Smoothing across ages is implemented analogously, but only for flows such as χ. For example, for excess-mortality hazard χ, for cohort grid point c, for age grid points a_j, a_{j+1}, a_{j+2}, the log-prior is equal to

$$-\frac{1}{\sigma}\Bigg[\frac{\log(\chi(a_{j+2}, c+a_{j+2})) - \log(\chi(a_{j+1}, c+a_{j+1}))}{a_{j+2}-a_{j+1}} - \frac{\log(\chi(a_{j+1}, c+a_{j+1})) - \log(\chi(a_j, c+a_j))}{a_{j+1}-a_j}\Bigg]^2,$$

where σ is the prior on second-order smoothing of χ with respect to age.

Smoothing across age and cohort is also implemented with an analogous approximation of the cross derivative. For an example again with excess-mortality hazard χ, for cohort grid points c_k, c_{k+1} and age grid points a_j, a_{j+1}, the log-prior is equal to

$$-\frac{1}{\sigma}\Bigg[\log(\chi(a_{j+1}, c_{k+1}+a_{j+1})) - \log(\chi(a_{j+1}, c_k+a_{j+1})) - \log(\chi(a_j, c_{k+1}+a_j)) - \log(\chi(a_j, c_k+a_j))\Bigg]^2 \Big/ [(a_{j+1}-a_j)(c_{k+1}-c_k)],$$

where σ is the prior on smoothing of χ with respect to age and cohort.

The model and smoothing priors are combined with a data likelihood to produce a posterior distribution on model parameters. The data likelihood is an offset lognormal model, where each observation i is coded as a triple (v_i, s_i, z_i), and using I_i to denote the integrand of the model parameters corresponding to this observation, observation i contributes the following to the log-likelihood:

$$-\log(2\pi s_i^2)/2 - \left(\frac{\log(I_i+z_i) - \log(v_i+z_i)}{2s_i^2}\right)^2.$$

The model is fit by finding the maximum a posteriori value for the parameters, using the Ipopt system for constrained nonlinear optimization.

2 Specialized/Simplified for CHD

Since CHD is a congenital condition, in all of the modeling in the present paper the DisMod-PDE model can be simplified to a one-compartment ODE. The first step in this simplification is to constrain the incidence and remission rates to be zero for all ages and times (i.e. $\iota(a,t) = \rho(a,t) = 0$). This removes all flow between compartments, and simplifies the differential equations to the following

$$\frac{dS(a+\tau, t+\tau)}{d\tau} = -\omega S;$$
$$\frac{dC(a+\tau, t+\tau)}{d\tau} = -(\omega + \chi)C.$$

Since the data we have available is either a measurement of CHD prevalence or of CHD cause-specific mortality rate, the integrands that appear as I_i in the model likelihood are $C/(S+C)$ and $\chi \cdot C/(S+C)$. We may further simplify the model for a rare condition like recalled CHD (with prevalence less than 0.5%) by constraining $\omega = 0$ and $S = 1$, which introduces an inaccuracy of less than 1% in the integrand $C/(S+C)$. This further simplified the differential equations to a single ODE

$$\frac{dC(a+\tau, t+\tau)}{d\tau} = -\chi(a,t)C(a,t).$$

Since we assumed that birth prevalence was constant over time, there is a single parameter C_0 which defines the initial conditions $C(0,t) = C_0$. For $\chi(a,t)$ we used knots in age at ages $(0, 1, 2, 3, 4, 5, 10, 15, \ldots, 65)$ and knots in cohorts at 5 year age intervals from 1900 to 2020, as well as knots at 2050 and 2100 for extrapolation.

The stock C was smoothed across cohorts with $\sigma = 1$, and the excess mortality χ was smoothed across ages, cohorts, and age/cohort with $\sigma = 1$ as well. For knots outside the time period where data was available (i.e. before 1968 and after 2010), we constrained χ to be constant in cohort, meaning $\chi(a, t-1) = \chi(a,t)$.

For each age-/time-specific prevalence measurement, we calculated the value v_i as the survey-weighted mean from National Health Interview Survey, and for the standard deviation s_i, we used $\sqrt{0.003/n_i}$, where n_i is the number of respondents in the survey for the given age and time and 0.003 is a rough estimate of the prevalence of CHD (per one). We took the offset $z_i = 1$ to make the error distribution roughly Gaussian.

For each age-/time-specific excess-mortality measurement, we calculated the value v_i as the number of cause-specific deaths in Multiple-Cause Mortality Files divided by the population from the Human Mortality Database. We set standard deviation $s_i = 0.05$ and offset $z_i = 10^{-6}$, which makes the error distribution roughly log-normal, with around 5% relative deviations from the observed data allowed.

To generate uncertainty intervals for our estimates, we used a parametric bootstrap procedure to resample each prevalence measurements from a binomial

distribution $\text{Bi}(n_i, v_i)$ with parameter n_i equal to the number of respondents in the survey for this measurement and parameter v_i equal to the survey-weighted mean of responses. We repeated this procedure $1,000$ times and reported the 2.5- and 97.5-th percentile values as our 95% uncertainty intervals.

We fit the model separately for males and females.

References

[1] Bell, B. M. and Flaxman, A. D. (2013). A Statistical Model and Estimation of Disease Rates as Functions of Age and Time. *SIAM Journal on Scientific Computing*, 35(2), B511-B528.
