# Supplementary material for: Projected growth of the adult congenital heart disease population in the United States to 2050: an integrative systems modeling approach
Source: Popul Health Metr. 2015 Oct 15;13:29. doi: 10.1186/s12963-015-0063-z (PMC4606959; doi:10.1186/s12963-015-0063-z)
Supplement: Additional file 3: — Sensitivity analysis of ACHD DisMod-PDE parameters. (PDF 94 kb) [file 12963_2015_63_MOESM3_ESM.pdf]

Online supplementary file 2: Sensitivity analysis of ACHD DisMod-PDE parameters

Abraham D. Flaxman

This online supplementary file provides a sensitivity analysis for the DisMod-PDE model used for estimating prevalence of Adult Congenital Heart Disease (ACHD). It focuses on the main estimates of the paper, the size of the ACHD population in the United States over time.

1 Hold-out cross validation

To test the sensitivity of our model predictions, we consider holdout scenarios where we limit the model to include only data from before a certain year. By comparing our estimates of ACHD cases based on all available data to estimates with progressively less data, we can obtain some indication of how additional data may change our projection going forward.

We consider holding out scenarios that include only data before the following years: 2009, 2007, 2005, and 2000. This changes estimated number of ACHD cases by -8% to 10%, sometimes increasing and sometimes decreasing year-specific estimates of the ACHD population size. Tables 1-4 show the comparisons.

	1968	2010	2025	2050
Baseline Analysis (Thousands)	117.9	272.9	355.4	509.7
Without data after 2009 (Thousands)	123.3	282.6	366.7	520.0
Relative Error (%)	4.6	3.5	3.2	2.0

Table 1: Sensitivity analysis comparing baseline estimate to estimate generated by including only data from before 2009.

2 Sensitivity of model estimates to prior

The DisMod-PDE model has a number of parameters and the modeling process includes a number of additional choices. We have attempted to use "weakly informative" priors in our design, so that the results are influenced primarily by the data. To investigate how sensitive our estimates are to these choices, we

	1968	2010	2025	2050
Baseline Analysis (Thousands)	117.9	272.9	355.4	509.7
Without data after 2007 (Thousands)	113.0	262.2	342.3	489.3
Relative Error (%)	-4.2	-3.9	-3.7	-4.0

Table 2: Sensitivity analysis comparing baseline estimate to estimate generated by including only data from before 2007.

	1968	2010	2025	2050
Baseline Analysis (Thousands)	117.9	272.9	355.4	509.7
Without data after 2005 (Thousands)	124.3	284.3	365.8	535.3
Relative Error (%)	5.5	4.2	2.9	5.0

Table 3: Sensitivity analysis comparing baseline estimate to estimate generated by including only data from before 2005.

	1968	2010	2025	2050
Baseline Analysis (Thousands)	97.8	250.6	338.4	483.2
Without data after 2000 (Thousands)	90.4	235.4	324.4	508.2
Relative Error (%)	-7.6	-6.1	-4.1	5.2

Table 4: Sensitivity analysis comparing baseline estimate to estimate generated by including only data from before 2000.

compared the baseline estimates to estimates for larger and smaller values of each of the priors.

For the with-condition compartment $C(a,t)$, we used a second-order smoothing prior across cohorts, as described precisely in Online supplementary file 1 (and originally in [1]). In the notation there, for our baseline model we took $\sigma = 1$. To understand the influence of this choice, we considered smoothing with σ 10 times larger and 10 times smaller. When $\sigma = 0.1$, the prior is more informative and the estimates are compressed (and shifted up), yielding estimates that are 3% higher than baseline in early years and 2% higher in future predictions. When $\sigma = 10$, the priors is less informative and the estimates are nearly identical, differing for all years by less than 0.1%. This demonstrates that our baseline prior is sufficiently diffuse when smoothing C across cohorts, and also that a substantially stronger prior on smoothness would change the forecast by less than 5%. See Tables 5 and 6 for a full comparison.

For the excess mortality rate $\chi(a,t)$, we used second-order smoothing priors across cohorts and ages, as well as a cross-smoothing prior across age and cohort, as described in Online supplementary file 1. For our baseline model we took $\sigma = 1$ for cohort smoothing, age smoothing, and cross smoothing. To understand the influence of these choices, we considered smoothing with larger and smaller

	1968	2010	2025	2050
Baseline Analysis, $\sigma = 1$ (Thousands)	117.9	272.9	355.4	509.7
C cohort smoothing, $\sigma = 0.1$ (Thousands)	121.5	279.6	362.6	518.5
Relative Error (%)	3.1	2.4	2.0	1.7

Table 5: Sensitivity analysis comparing baseline estimate with C cohort $\sigma = 1$ to estimate generated with more informative prior on cohort smoothing (C cohort $\sigma = 0.1$).

	1968	2010	2025	2050
Baseline Analysis, $\sigma = 1$ (Thousands)	117.9	272.9	355.4	509.7
C cohort $\sigma = 10$ (Thousands)	117.8	272.9	355.4	509.6
Relative Error (%)	-0.0	-0.0	-0.0	-0.0

Table 6: Sensitivity analysis comparing baseline estimate with C cohort $\sigma = 1$ to estimate generated with lesss informative prior on cohort smoothing (C cohort $\sigma = 10$).

values of σ one at a time.

When $\chi(a,t)$ has cohort smoothing of $\sigma = 0.1$, the prior is more informative and the estimates are compressed (and shifted up), yielding estimates that are 9% higher than baseline in early years and 5% higher in future predictions. When $\sigma = 10$, the priors is less informative and the estimates are nearly identical, differing for all years by at most 0.1%. This demonstrates that our baseline prior is sufficiently diffuse when smoothing χ across cohorts, and also that a substantially stronger prior on smoothness would change the forecast by 5-9%. See Tables 7 and 8 for a full comparison.

	1968	2010	2025	2050
Baseline Analysis $\sigma = 1$ (Thousands)	117.9	272.9	355.4	509.7
χ cohort $\sigma = 0.1$ (Thousands)	128.3	292.4	376.6	535.4
Relative Error (%)	8.9	7.1	6.0	5.0

Table 7: Sensitivity analysis comparing baseline estimate with χ cohort smoothing with $\sigma = 1$ to estimates generated with more informative prior on cohort smoothing (χ cohort smoothing of $\sigma = 0.1$).

When $\chi(a,t)$ has more informative *age* smoothing of $\sigma = 0.1$, the prior is strong enough to change the estimates more substantially, yielding estimates that are 56% lower than baseline in early years and 36% lower in future predictions. However, the sensitivity to this prior is considerably less pronounced at larger values of σ. For example, when the $\sigma = 0.9$, the prior is slightly more informative than in the baseline scenario, and the estimates are only 1-2% lower.

	1968	2010	2025	2050
Baseline Analysis, $\sigma = 1$ (Thousands)	117.9	272.9	355.4	509.7
χ cohort smoothing, $\sigma = 10$ (Thousands)	117.7	272.6	355.1	509.3
Relative Error (%)	-0.1	-0.1	-0.1	-0.1

Table 8: Sensitivity analysis comparing baseline estimate with χ cohort smoothing with $\sigma = 1$ to estimates generated with less informative prior on cohort smoothing (χ cohort smoothing of $\sigma = 10$).

When $\sigma = 10$, the priors is less informative and the estimates are larger, differing by 6% in 1968 and 3% in 2050. This demonstrates that our baseline prior is sufficiently diffuse when smoothing χ across ages, and also that a substantially stronger prior on smoothness would compress the age pattern and substantially the forecast (by 36%). See Tables 9–11 for a full comparison.

	1968	2010	2025	2050
Baseline Analysis, $\sigma = 1$ (Thousands)	117.9	272.9	355.4	509.7
χ age smoothing, $\sigma = 0.1$ (Thousands)	51.6	143.4	207.6	327.9
Relative Error (%)	-56.2	-47.5	-41.6	-35.7

Table 9: Sensitivity analysis comparing baseline estimate with χ age smoothing with $\sigma = 1$ to estimates generated with more informative prior on age smoothing (χ age smoothing of $\sigma = 0.1$).

	1968	2010	2025	2050
Baseline Analysis, $\sigma = 1$ (Thousands)	117.9	272.9	355.4	509.7
χ age smoothing, $\sigma = 0.9$ (Thousands)	116.1	269.8	352.0	505.6
Relative Error (%)	-1.5	-1.2	-1.0	-0.8

Table 10: Sensitivity analysis comparing baseline estimate with χ age smoothing with $\sigma = 1$ to estimates generated with slightly more informative prior on age smoothing (χ cohort smoothing of $\sigma = 0.9$).

When $\chi(a, t)$ has more informative *cross* smoothing of $\sigma = 0.1$, the estimates are 1-2% larger, and when the cross smoothing $\sigma = 10$ the estimates are nearly identical. See Tables 12–13 for a full comparison.

3 Sensitivity to including birth prevalence measurements from Atlanta surveillance

One of the most detailed sources of information on the birth prevalence of CHD comes from an analysis of data collected by the Metropolitan Atlanta Congenital

	1968	2010	2025	2050
Baseline Analysis, $\sigma = 1$ (Thousands)	117.9	272.9	355.4	509.7
χ age smoothing, $\sigma = 10$ (Thousands)	125.0	286.0	369.6	526.4
Relative Error (%)	6.1	4.8	4.0	3.3

Table 11: Sensitivity analysis comparing baseline estimate with χ age smoothing with $\sigma = 1$ to estimates generated with less informative prior on age smoothing (χ age smoothing of $\sigma = 10$).

	1968	2010	2025	2050
Baseline Analysis, $\sigma = 1$ (Thousands)	117.9	272.9	355.4	509.7
χ cross smoothing, $\sigma = 0.1$ (Thousands)	120.7	278.0	360.9	516.0
Relative Error (%)	2.4	1.9	1.5	1.2

Table 12: Sensitivity analysis comparing baseline estimate with χ cross smoothing with $\sigma = 1$ to estimates generated with more informative prior on cross smoothing (χ cross smoothing of $\sigma = 0.1$).

	1968	2010	2025	2050
Baseline Analysis, $\sigma = 1$ (Thousands)	117.9	272.9	355.4	509.7
χ cross sigma, $\sigma = 10$ (Thousands)	117.8	272.9	355.4	509.6
Relative Error (%)	-0.0	-0.0	-0.0	-0.0

Table 13: Sensitivity analysis comparing baseline estimate with χ cross smoothing with $\sigma = 1$ to estimates generated with less informative prior on cross smoothing (χ age smoothing of $\sigma = 10$).

Defects Program from 1998-2005 [2]. In this study, the authors found that out of 398,140 births, there were 3240 infants with CHD, for an overall birth prevalence of 8.14 per 1,000. The authors also found that the birth prevalence of critical CHD was 1.56 per 1,000.

As an additional investigation into the sensitivity of our results to the definition of recalled CHD, we calculated two alternative estimates of ACHD prevalence, one with the additional measurement of birth prevalence of 8.14 per 1,000 for 1998-2005, and the other with a measurement of birth prevalence 1.56 per 1,000.

For each scenario, we took the measurement to have relative error to match a rate calculated from a binomial distribution with 398,140 trials and success probability equal to the measured prevalence of the scenario (i.e. 1.8% relative error for the 8.14 per 1,000 scenario and 4.0% for the 1.56 per 1,000 scenario).

We found that including the 8.14 per 1,000 birth prevalence substantially raises our estimates, e.g for 2010, the data raises the estimate from our baseline estimate of 273,000 ACHD cases to a high estimate of 868,000 ACHD cases,

constituting a 3.2-fold increase. On the other hand, including the 15.6 per 10,000 birth prevalence of critical CHD lowers the estimate to 154,000 critical ACHD cases, a 2.1-fold decrease. Tables 14 and 15 compare these estimates over a range of times.

	1968	2010	2025	2050
Baseline Analysis (Thousands)	117.9	272.9	355.4	509.7
Birth prevalence of 8.14 (Thousands)	446.0	867.7	953.1	1,212.8

Table 14: Sensitivity analysis comparing baseline estimate to estimate generated by including birth prevalence of CHD measured at 8.14 per 1,000 in Metropolitan Atlanta Congenital Defects Program from 1998-2005.

	1968	2010	2025	2050
Baseline Analysis (Thousands)	117.9	272.9	355.4	509.7
Birth prevalence of 1.56 (Thousands)	48.9	129.3	182.5	287.9

Table 15: Sensitivity analysis comparing baseline estimate to estimate generated by including birth prevalence of critical CHD measured at 1.56 per 1,000 in Metropolitan Atlanta Congenital Defects Program from 1998-2005.

From this, we can conclude that recalled ACHD is probably capturing more than just the critical CHD cases, but is far from capturing all CHD cases, and the model is rather sensitive to the prevalence data available. This further confirms the importance of prevalence data that is demonstrated by the uncertainty quantification included in the main paper.

4 Sensitivity to a scenario where birth prevalence is decreasing over time

A truism commonly attribute to Yogi Berra is that, "it's tough to make predictions, especially about the future." Indeed. This section considers the sensitivity of our predictions to the possibility that prenatal screening for CHD drives an increasing secular trend in the rate of termination after prenatal diagnosis. To investigate the impact of this possible trend, we created scenarios where sex-specific prevalence matched the baseline scenario until 2015, and then began to decline annually by a fixed percentage, of either 1%, 3%, or 10% per year. As you would expect, we found that a secular trend of decreasing birth prevalence produced a decreased projection of the adult population, as compared to the baseline projection. Tables 16–18 show the sensitivity of the projections to this scenario.

	1968	2010	2025	2050
Baseline Analysis (Thousands)	117.9	272.9	355.4	509.7
1% annual decline (Thousands)	116.9	271.1	353.5	493.0
Relative Error (%)	-0.8	-0.7	-0.6	-3.3

Table 16: Sensitivity analysis comparing baseline estimate to estimate generated by a secular trend decreasing birth prevalence of CHD by 1% per year starting in 2015.

	1968	2010	2025	2050
Baseline Analysis (Thousands)	117.9	272.9	355.4	509.7
3% annual decline (Thousands)	116.9	271.1	353.5	471.0
Relative Error (%)	-0.8	-0.7	-0.6	-7.6

Table 17: Sensitivity analysis comparing baseline estimate to estimate generated by a secular trend decreasing birth prevalence of CHD by 3% per year starting in 2015.

	1968	2010	2025	2050
Baseline Analysis (Thousands)	117.9	272.9	355.4	509.7
10% annual decline (Thousands)	116.9	271.1	352.1	317.0
Relative Error (%)	-0.9	-0.7	-0.9	-37.8

Table 18: Sensitivity analysis comparing baseline estimate to estimate generated by a secular trend decreasing birth prevalence of CHD by 10% per year starting in 2015.

5 Sensitivity to alternative population forecasts

In our baseline estimate, we incorporated no uncertainty in our population forecast, and simply used the US Census Department 2012 national projection as if is has perfect precision. To understand the sensitivity of our estimates to this assumption, in this section, I compare the baseline projection to the US Census Department 2012 High and Low Series projections, which assume more or less in-migration than the Middle Series used in the baseline. Tables 19 and 20 show the results.

References

[1] Bell BM and Flaxman AD. A Statistical Model and Estimation of Disease Rates as Functions of Age and Time. *SIAM Journal on Scientific Computing.* 2013;35(2):B511-B528.

	1968	2010	2025	2050
Baseline Analysis (Thousands)	117.9	272.9	355.4	509.7
High Series (Thousands)	117.9	272.9	359.2	534.2
Relative Error (%)	0.0	0.0	1.1	4.8

Table 19: Sensitivity analysis comparing baseline estimate to estimate generated by the US Census Department 2012 High Series projection.

	1968	2010	2025	2050
Baseline Analysis (Thousands)	117.9	272.9	355.4	509.7
Low Series (Thousands)	117.9	272.9	351.7	485.2
Relative Error (%)	0.0	0.0	-1.1	-4.8

Table 20: Sensitivity analysis comparing baseline estimate to estimate generated by US Census Department 2012 Low Series projection.

[2] Reller MD, Strickland MJ, Riehle-Colarusso T, Mahle WT, Correa A. Prevalence of Congenital Heart Defects in Metropolitan Atlanta, 19982005. *The Journal of Pediatrics.* 2008;153(6):807-813. doi:10.1016/j.jpeds.2008.05.059.
